# Outcome indicators for cross linking in pediatric keratoconus

**DOI:** 10.3389/fmed.2023.1149641

**Published:** 2023-05-12

**Authors:** Denise Wajnsztajn, Or Shmueli, Yehuda Tarnovsky, Joseph Frucht-Pery, Abraham Solomon

**Affiliations:** Department of Ophthalmology, Hadassah-Hebrew University Medical Center, Jerusalem, Israel

**Keywords:** keratoconus, cross-linking, outcome indicators, predictive factors, efficacy, pediatric

## Abstract

**Purpose:**

To evaluate the predictive factors for successful corneal collagen cross-linking (CXL) in pediatric patients with Keratoconus (KC).

**Methods:**

This retrospective study was conducted using a prospectively built database. Patients (18 years old or younger) underwent CXL for KC between 2007 and 2017, with a 1-year follow-up period or longer. The outcomes included changes in Kmax (delta [Δ] Kmax = Kmax_last_ − Kmax_pre_) and LogMAR visual acuity (ΔLogMAR = LogMAR_last_ − LogMAR_pre_).

The effects of CXL type (accelerated or non-accelerated), demographics (age, sex, background of ocular allergy, ethnicity), preoperative LogMAR visual acuity, maximal corneal power (Kmax), pachymetry (CCT_pre_), refractive cylinder, and follow-up (FU) time on the outcomes were analyzed.

**Results:**

One hundred thirty-one eyes of 110 children were included (mean age, 16 ± 2 years; range, 10–18 years). Kmax and LogMAR improved from baseline to last visit: from 53.81 D ± 6.39 D to 52.31 D ± 6.06 D (*p* < 0.001) and from 0.27 ± 0.23 LogMAR units to 0.23 ± 0.19 LogMAR units (*p* = 0.005), respectively. A negative ΔKmax (meaning corneal flattening) was associated with a long FU, low CCT_pre_, high Kmax_pre_, high LogMAR_pre,_ and non-accelerated CXL on univariate analysis. High Kmax_pre_ and non-accelerated CXL were associated with negative ΔKmax in the multivariate analysis.

A negative ΔLogMAR (meaning vision improvement) was associated with a high LogMAR_pre_ in univariate analysis.

**Conclusion:**

CXL is an effective treatment option in pediatric patients with KC. Our results showed that the non-accelerated treatment was more effective than the accelerated treatment. Corneas with advanced disease had a greater effect on CXL.

## Introduction

Keratoconus (KC) is a progressive corneal ectasia characterized by progressive central or paracentral thinning, protrusion, and irregular astigmatism, with the potential for severe visual loss. KC onset occurs between the first decade of life and puberty. Younger patients, especially young males (1), often present with more advanced and severe disease, a rapidly progressive course, and significant asymmetry (2–4). Advanced KC causes significant visual impairment and is of particular concern to this population because it may critically impact young patients’ social, educational, and professional development.

Corneal collagen cross-linking (CXL) is used to strengthen the cornea by photochemically creating new covalent bonds within and between amino acid residues in the collagen fibers of the cornea through a combination of vitamin B_2_ (riboflavin) and longer-wavelength ultraviolet A radiation (370 nm). This increases the biomechanical strength of the keratoconic cornea and halts ectasia progression of ectasia (5).

To date, the maximum corneal power (K_max_) and visual acuity (measured as the minimum angle of resolution [logMAR]) are acceptable parameters for evaluating CXL efficacy and safety, respectively (6). The success of CXL in pediatric patients is conflicting. While some studies agree that CXL in this population can stop KC progression (7–13), others claim that this treatment is insufficient or only partially effective, showing disease progression in up to 55% of treated eyes (14–19).

The main shortcomings of the current literature on pediatric CXL are the small number of studies with more than 100 eyes and short follow-up times, with only one randomized controlled study to date (20). Moreover, most studies compared final outcomes to baseline preoperative measures, and there is a lack of studies that systematically evaluated the factors predicting CXL success using a multivariate analysis approach (21).

Our study used multivariate analysis to evaluate long-term outcomes and preoperative predictors of successful CXL treating KC in children.

## Materials and methods

### Study design and patient selection

This was a retrospective cohort study. We collected data from patients with KC treated with CXL between 2007 and 2017 at the Department of Ophthalmology Cornea Service of Hadassah-Hebrew University Medical Center (Jerusalem, Israel).

This study adhered to the principles of the Declaration of Helsinki. Approval was obtained from the Institutional Review (IRB)Board/Ethics Committee (approval number 18-0221).

The included patients were aged 18 years or younger at the time of treatment and underwent CXL for progressive KC. Informed consent was obtained from the legal guardian (aged <18 years), allowing for treatment. The IRB waived the requirement for informed consent for participation in this study owing to the retrospective and anonymous nature of data analysis.

KC was diagnosed based on topographic features (EyeSys 2000; EyeSys Vision Inc., Houston, Texas, USA) (22).

Progression of KC was defined as an increase of at least 1.00 D in Kmax or in the refractive cylinder within 1 year or a patient’s report of deteriorating visual acuity without any other underlying cause in cases where previous refraction or topographic assessment was not possible.

Before CXL, we performed a complete eye examination with anterior segment evaluation, intraocular pressure measurement, Schirmer test, and central corneal thickness measurement [CCT (Corneo-Gage Plus™, Sonogage, Cleveland, OH, USA)]. Any abnormalities were managed before CXL was performed.

Follow-up lasted for at least 1 year after CXL treatment.

Exclusion criteria included insufficient follow-up time (less than 1 year after CXL) and insufficient critical data for calculating primary outcomes (e.g., lack of Kmax_pre_ measurement).

CXL was not performed in patients with active ocular surface disease, a history of herpes, stable KC, or pregnant women.

### Corneal cross-linking procedure

All CXL procedures involve epithelium-off (epi-off). After the application of topical anesthetics, a slit lamp-assisted corneal abrasion of 8 mm diameter was made using a blunt spatula, and isotonic [0.1% riboflavin, 20% dextran (MedioCROSS D, Avedro)] or hypotonic (0.1% riboflavin (MedioCROSS H, Avedro) in corneas <400 μm) riboflavin was applied every 3 min for 30 min. Pachymetry was performed to ensure CCT > 400 μm before UVA exposure. None of the patients in our study required general anesthesia.

The cornea was subsequently exposed to a 3-mW/cm^2^ 365-nm UV light source for 30 min in the non-accelerated (Dresden) protocol, or to a 9-mW/cm^2^ 365-nm UV light source for 10 min in the accelerated protocol (UV-X ™ Specifications, IROC, Zurich, Switzerland). In both protocols, a total of 5.4 J/cm^2^ energy was delivered.

Subsequently, a 17 mm soft bandage contact lens (BCL; Sophlex, Israel) was placed on the cornea for at least 7 days or until complete re-epithelialization was evident. The patients received topical 0.1% dexamethasone and 0.3% gentamycin three and four times a day, respectively, for a week. After 7 days or when complete epithelialization was evident, BCL was removed, and topical fluorometholone 0.1% was applied three times a day for an additional 3 months.

Follow-up examinations were performed at 1, 3, 6, and 12 months of the first year and then yearly, including topography, uncorrected and corrected distance visual acuity (CDVA), and manifest refraction.

### Data collection

We retrospectively recorded data from a prospectively built database of patients who underwent CXL for KC at the Cornea Service of The Ophthalmology Department of Hadassah Medical Center. The predictive factors documented included age at treatment, gender, a background of ocular allergy (diagnosis of allergic keratoconjunctivitis, vernal keratoconjunctivitis (VKC), undefined ocular allergy, or significant papillary reaction), ethnicity (Arabic or Jewish origin), follow-up period (in months), CCT at treatment (CCT_pre_, in microns), accelerated or non-accelerated (Dresden) protocol, Kmax before treatment (Kmax_pre,_ in diopters), CDVA in LogMAR before treatment (LogMAR_pre_), and refractive cylinder before treatment (Cyl_pre_, in diopters).

Data were collected between 2007 and 2017. Visual acuity was tested using a Snellen chart and was converted to a logMAR visual acuity score. Cyl was extracted from manifest refraction, and CCT was obtained using the Corneo-Gage Plus™. The maximal corneal power (Kmax) was obtained using EyeSys through an axial numeric map provided by the topographer. The highest values were recorded and used for comparison (23).

### Data analysis

The two study outcome measures were changes in Kmax (Delta Kmax) and visual acuity (Delta LogMAR) after CXL.

Delta Kmax was defined as the difference between the maximal corneal power (in diopters) at the last follow-up (Kmax_last_) and the maximal corneal power before CXL (Kmax_pre_): Delta Kmax = Kmax_last_-Kmax_pre_.

Delta logMAR was defined as the difference in logMAR CDVA between the last follow-up (LogMAR_last_) and logMAR before treatment (LogMAR_pre_): Delta LogMAR = LogMAR_last_ − LogMAR_pre_.

A value of 0 or negative values in Delta Kmax or Delta LogMAR indicated no decrease (stabilization) or improvement in Kmax or CDVA, respectively, demonstrating a favorable effect of CXL.

Delta Kmax and Delta LogMAR were analyzed using paired Student’s *t*-tests to evaluate the significance of the changes.

We first tested the effects of the independent variables on the outcome measures (Delta Kmax and Delta LogMAR) using a univariate analysis. We used the paired t-test, Mann–Whitney test and Pearson’s correlation (with r coefficient) for continuous variables, including age, LogMAR_pre_, Kmax_pre_, CCT_pre_, Cyl_pre_, and follow-up time. We used the chi-squared test for categorical variables, including accelerated or non-accelerated CXL, sex, ocular allergic background, and ethnicity.

Subsequently, variables that demonstrated a significant effect on either outcome measure in the univariate analysis were included in the multivariate analysis using stepwise linear regression.

*p* values < 0.05 were considered statistically significant.

Data were analyzed using SPSS version 24.0 (IBM).

## Results

We included 131 eyes of 110 patients. The mean follow-up was 32.76 ± 21.03 months (range: 12–104.47 months). The baseline patient characteristics are presented in Table 1. Hypotonic riboflavin was applied before CXL in 10 eyes with CCT < 400 μm. All these eyes except for two achieved a minimal CCT of 400 μm after the instillation of hypotonic riboflavin before UVA treatment. These two eyes had CCT of 380 and 397 μm, respectively. Treatment was performed because of the potentially high risk of vision loss due to advanced KC. There were no complications in these eyes, including corneal edema or scarring.

**Table 1:** Baseline patient’s characteristics.

Baseline continuous variables			
Variables	^**1**^ ***N***	Median	Mean ± ^2^SD (Min–Max)
Age (years)	131	16.00	15.71 ± 2.07 (10; 18)
Follow up (months)	131	24.97	32.76 ± 21.03 (12.0; 104.47)
^3^Pachymetry (microns)	128	470.00	464.78 ± 44.71 (328–575)
^4^Kmax_pre_ (D)	131	52.40	53.81 ± 6.39 (42.70–70.10)
^5^LogMAR_pre_	129	0.22	0.27 ± 0.23 (0–1.96)
^6^Cyl_pre_ (D)	120	−3.25	−4.11 ± 3.00 (−12.50; 0)
Baseline categorical variables			
Accelerated/non-accelerated	*N* = 131	Non-accelerated	41 eyes
		Accelerated	90 eyes
Gender	*N* = 131	Male	102 eyes
		Female	29 eyes
Ethnicity	*N* = 128	Jewish	101 eyes
		Arabic	27 eyes
Allergy	*N* = 131	Allergy	35 eyes
		No allergy	96 eyes
Baseline characteristics of accelerated\non-accelerated treatment groups			
Variable	Non-accelerated	Accelerated	*p*-value
Age (years)	16.22	15.48	0.057
Follow up (months)	55.23	23.02	**<0.001** **
Pachymetry (microns)	453.90	469.55	0.068
Kmax_pre_	56.4810	52.5900	**0.001**
LogMAR_pre_	0.3387	0.2359	**0.018**
Cyl_pre_ (D)	−4.016	−4.145	0.810
Sex (male)	80.5%	76.7%	0.625
Allergy	14.6%	32.2%	**0.035**
Ethnicity (Jewish)	71.1%	82.2%	0.157

Continuous variables are presented by Mean, median, standard deviation and minimum/maximum values. Comparison of baseline characteristics of accelerated\non-accelerated treatment groups was analyzed with *t*-test and Chi^2^ test, respectively. Bold values are for *p*-values of significance.

1 *N* = number of eyes with available data.

2 SD = standard deviation.

3 Pachymetry was measured before corneal epithelial removal.

4 Kmax_pre_ = maximal corneal power before cross-linking.

5 LogMAR_pre_ = Logarithm of minimal angle of resolution before cross-linking.

6 Cyl_pre_ = refractive cylinder before cross-linking.

* *p* < 0.05.

** *p* < 0.01.

Annual post-CXL data were unavailable for all patients throughout follow-up; the number of patients and eyes for which data were available for analysis are indicated in the tables and figures below.

### Kmax and Delta Kmax outcome

A total of 131 eyes of 110 children had available Kmax results for a minimum of 1 year and were included in this analysis. Mean Kmax decreased from 53.81 D ± 6.39 D to 52.31 D ± 6.06 D (*p* < 0.001) at the last follow-up.

The mean Delta Kmax was −1.495 ± 2.946 D (median − 1.200).

Among the eyes, 52.6% had Delta Kmax ≤ −1D (improvement following treatment), 31.6% had Delta Kmax between −1D and +1D (stable), and 15.8% had Delta Kmax ≥ +1D (post-treatment progression).

Mean Delta Kmax values did not differ significantly between hypotonic (10 eyes; Mean Delta Kmax = −3.9) and isotonic (121 eyes; Mean Delta Kmax = −1.3) riboflavin pre-CXL instillation (*p* = 0.33; Mann–Whitney test).

### Delta Kmax: univariate analysis

**Figure 1:**
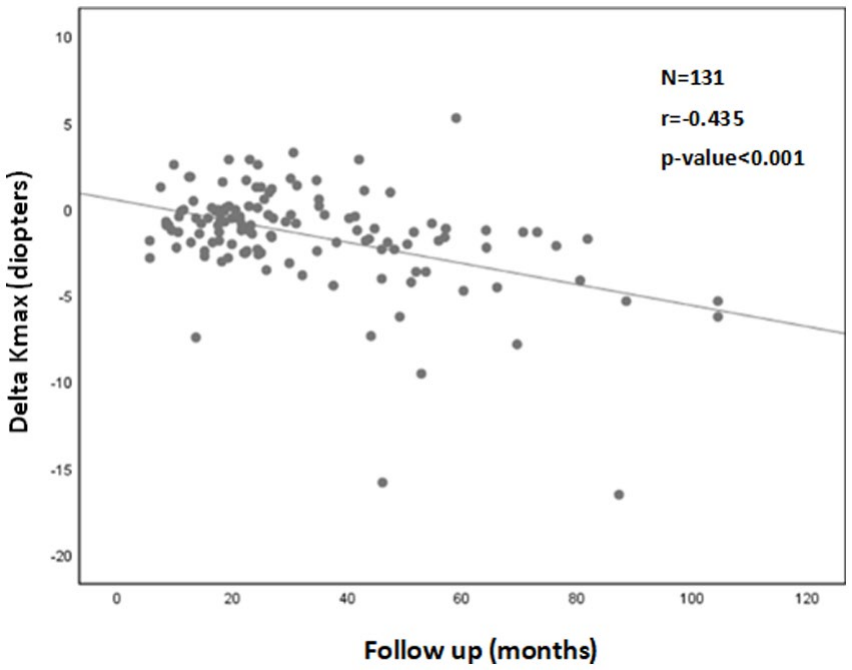
Correlation between follow-up time and ^1^Delta Kmax. More negative Delta Kmax values were correlated with longer follow-up times, indicating the treatment effect was greater with longer follow-up times. However, the correlation coefficient is low, with r^2^ = 0.19. Thus, although the correlation is significant, it is relatively weak. ^1^Delta Kmax = (maximal corneal power after cross-linking) − (maximal corneal power before cross-linking), measured in diopters. ^2^r = Pearson correlation coefficient.

Correlation analysis demonstrated a significant correlation between a longer follow-up time (r = −0.435; *p* < 0.001) and negative Delta Kmax values, indicating that the treatment effect was greater the further it was measured from the time of treatment (Figure 1).

**Figure 2:**
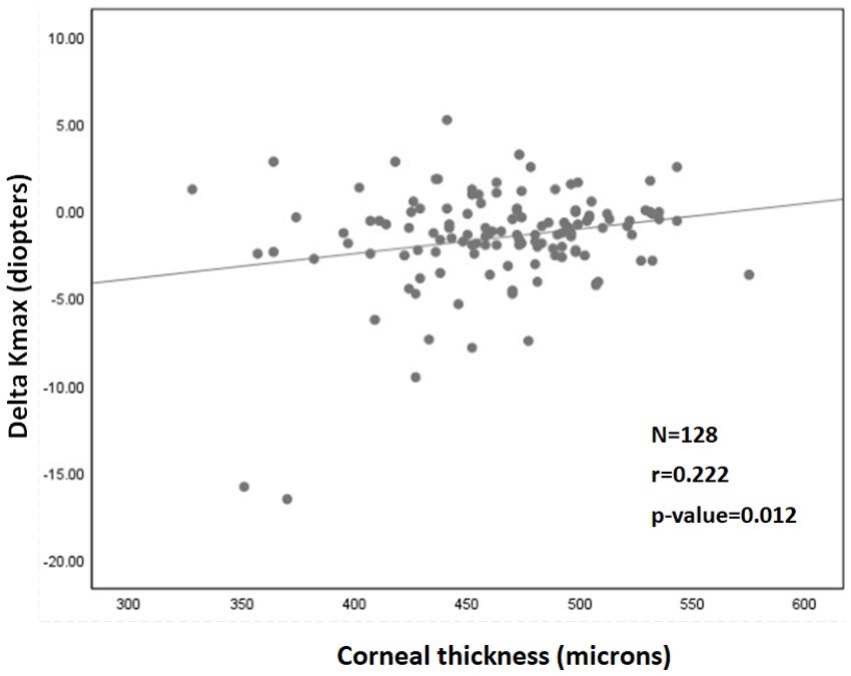
Correlation between corneal thickness (pachymetry) and ^1^Delta Kmax. More negative Delta Kmax values significantly correlated with thinner corneas, indicating that a thinner cornea can predict a better treatment outcome. However, the correlation coefficient is low, with r^2^ = 0.05. Thus, although the correlation is significant, it is relatively weak. ^1^Delta Kmax = (maximal corneal power after cross-linking) − (maximal corneal power before cross-linking), measured in diopters. ^2^r = Pearson correlation coefficient.

**Figure 3:**
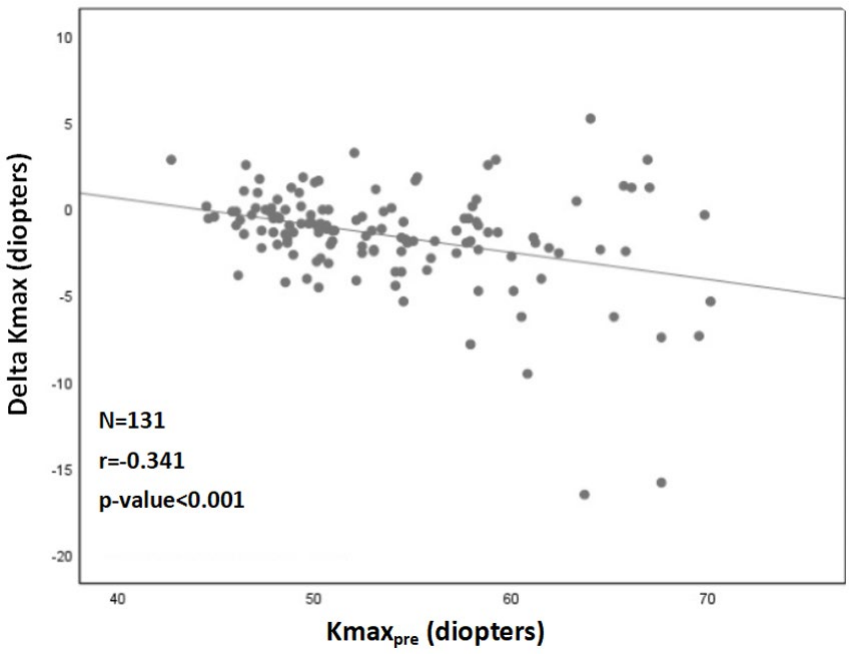
Correlation between ^1^Kmax_pre_ and ^2^Delta Kmax. More negative Delta Kmax values significantly correlated with higher Kmax_pre_ values (more severe baseline keratoconus). However, the correlation coefficient is low, with r^2^ = 0.13. Thus, although the correlation is significant, it is relatively weak. ^1^Kmax_pre_ = maximal corneal power before cross-linking. ^2^Delta Kmax = (maximal corneal power after cross-linking) − (maximal corneal power before cross-linking), measured in diopters. ^3^r = Pearson correlation coefficient.

A significant correlation was also found between a more negative Delta Kmax and thinner corneas (r = 0.222; *p* = 0.012; Figure 2), higher Kmax_pre_ (r = −0.341; *p* < 0.001; Figure 3), and higher LogMAR_pre_ values (r = −0.221; *p* = 0.012; the results of the correlation analysis are presented in Table 2).

**Table 2:** Analysis of variables affecting ^1^Delta Kmax.

Univariate analysis of continuous variables				
Variables	^2^ *N*	Pearson Correlation (^3^r)		*p*-value
Age	131	−0.018		0.842
Pachymetry	128	0.222		0.012*
^4^LogMAR_pre_	129	−0.221		0.012*
Follow-up time	131	−0.435		<0.001**
^5^Kmax_pre_	131	−0.341		<0.001**
^6^Cyl_pre_ (D)	120	0.170		0.065
Univariate analysis of categorical variables				
Variables	*N*	Mean	Median	*p*-value
Accelerated	90	−0.56 −3.55	−0.60 −2.40	<0.001^**^
Non-Accelerated	41			
Male	102	−1.46 −1.60	−1.20 −0.90	0.824
Female	29			
Jewish	101	−1.28 −2.14	−1.10 −1.30	0.177
Arab	27			
Allergy	35	−1.40 −1.53	−0.50 −1.25	0.824
No allergy	96			
Multivariate stepwise regression analysis				
Variables	*N*	*β*		*p*-value
Accelerated\ Non-accelerated	90 41	−2.453		<0.001^**^
Kmax_pre_	131	−0.096		**0.013**
LogMAR_pre_	129	−0.15		0.875
Follow-up time	131	−0.179		0.122
Pachymetry	128	−0.60		0.557
Model’s R^2^	**23.2%**			

Univariate analysis was done with correlation analysis for continuous variables and Chi-square test for categorical variables. Variables that were significant in the univariate analysis were included in multivariate analysis using stepwise approach linear regression.

1 Delta Kmax = (maximal corneal power after cross-linking) − (maximal corneal power before cross-linking).

2 *N* = number of patients.

3 r = Pearson correlation coefficient.

4 LogMAR_pre_ = Logarithm of minimal angle of resolution before cross-linking.

5 Kmax_pre_ = maximal corneal power before cross-linking.

6 Cyl_pre_ = refractive cylinder before cross-linking.

* *p* < 0.05.

** *p* < 0.01. Bold values are for *p*-values of significance.

**Figure 4:**
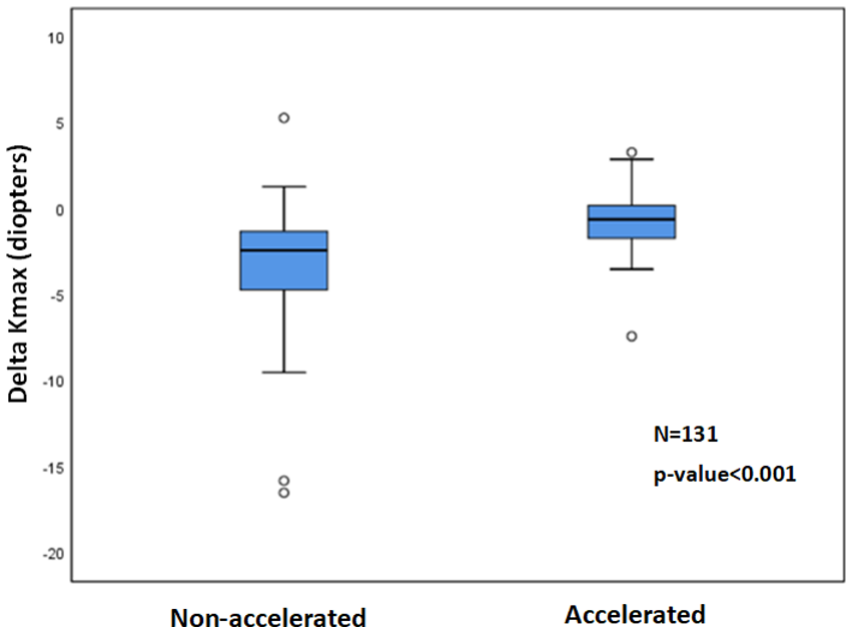
^1^Delta Kmax with non-accelerated and accelerated CXL^2^ protocols. The paired t-test showed significantly more mean negative Delta Kmax values with the non-accelerated protocol than with the accelerated protocol (*p* < 0.001), indicating a greater efficacy for the non-accelerated protocol. ^1^Delta Kmax = (maximal corneal power after cross-linking) − (maximal corneal power before cross-linking), measured in diopters. ^2^CXL = Cross-linking.

More negative Delta Kmax values were observed with the non-accelerated CXL protocol (*p* < 0.001) than with the accelerated CXL protocol (Figure 4).

Other variables had no significant effect on Delta Kmax (Table 2), including age (*p* = 0.842), sex (*p* = 0.824), ethnicity (Jewish or Arabic; *p* = 0.177), Cyl_pre_ (*p* = 0.065), and the presence of ocular allergy (p = 0.824).

The percentage of eyes with Delta Kmax ≤ −1 was 82.9% in the non-accelerated protocol group and 38.9% in the accelerated protocol group (*p* < 0.001; Table 3).

**Table 3:** Success rates of non-accelerated vs. accelerated cross-linking.

Variables	Non-accelerated	Accelerated	*p*-value
^1^Delta Kmax			
Delta Kmax ≤ −1	**82.9%**	38.9%	< 0.001**
+1 > Delta Kmax > −1	12.2%	**40%**	0.003**
Delta Kmax ≥ +1	4.9%	**21.1%**	0.019*
^2^Delta LogMAR			
Delta LogMAR ≤ −0.2	**24.4%**	7.1%	0.01*
+0.2 > Delta LogMAR > −0.2	60.1%	**86.9%**	0.008**
Delta LogMAR ≥ +0.2	14.6%	6%	0.17

Analysis was done with and Chi-square test.

1 Delta Kmax = (maximal corneal power after cross-linking) − (maximal corneal power before cross-linking).

2 Delta LogMAR = (LogMAR after cross-linking) − (LogMAR before cross-linking).

* *p* < 0.05.

** *p* < 0.01. Bold values are for *p*-values of significance.

### Delta Kmax: a multivariate analysis

Only two variables remained significant in the multivariate analysis (R^2^ = 23.2%; Table 2). In order of significance, these were accelerated/non-accelerated CXL protocols (β = −2.453; *p* < 0.001) and Kmax_pre_ (β = −0.096; *p* < 0.001). Together, these results indicate that the non-accelerated CXL protocol and a higher Kmax pre-CXL positively influenced corneal flattening following CXL treatment in pediatric patients with KC.

### LogMAR and Delta LogMAR outcome

A total of 123 eyes of 108 children had available LogMAR results for a minimum of 1 year and were included in this analysis. Mean LogMAR decreased from 0.27 ± 0.23 to 0.22 ± 0.19 (*p* = 0.005) at the last follow up. Mean Delta LogMAR was −0.05 ± 0.18 (median − 0.05).

**Figure 5:**
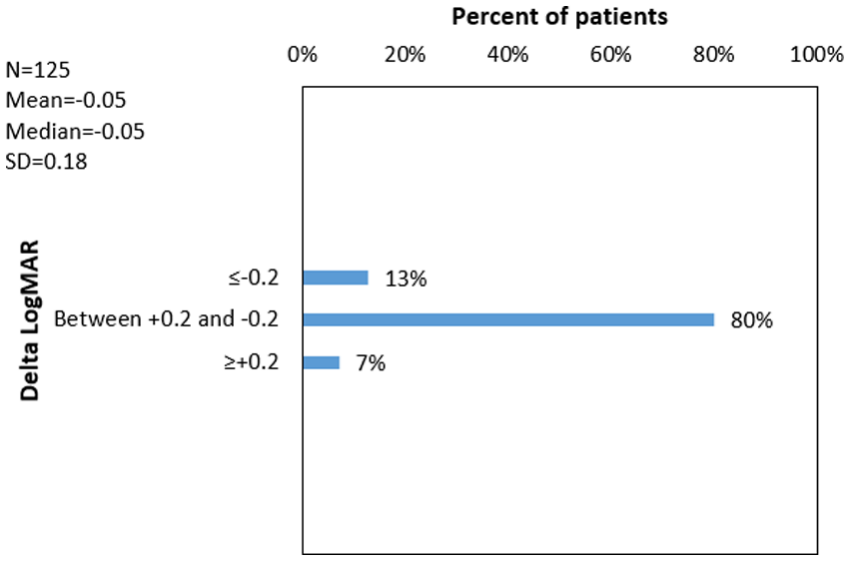
^1^Delta LogMAR distribution following cross-linking for Keratoconus. ^1^Delta LogMAR = (Logarithm of minimal angle of resolution after cross-linking) − (Logarithm of minimal angle of resolution before cross-linking). ^2^*N* = number of eyes with available data; ^3^SD = standard deviation.

12.8% of patients had Delta LogMAR ≤ −0.2 (improvement), 80% of patients had Delta LogMAR between −0.2 and +0.2 (stable), 7.2% of patients had Delta LogMAR ≥ +0.2 (worsen). Figure 5 shows the distribution of delta logMAR following CXL.

Mean Delta LogMAR values did not differ significantly between hypotonic (10 eyes; Mean Delta LogMAR = −0.13) and isotonic (113 eyes; Mean Delta Kmax = −0.04) riboflavin pre-CXL instillation (*p* = 0.77; Mann–Whitney test).

### Delta LogMAR: univariate analysis

The only variable found to be significantly correlated with a more negative Delta LogMAR (Table 4) was LogMAR_pre_ (r = −0.596; *p* < 0.001). Other variables were not significantly correlated with Delta LogMAR (Table 4), including age (*p* = 0.527), pachymetry (*p* = 0.853), follow-up time (*p* = 0.164), Kmax_pre_ (*p* = 0.712), Cyl_pre_ (*p* = 0.701), CXL protocol type (accelerated or non-accelerated; *p* = 0.350), sex (*p* = 0.663), ethnicity (*p* = 0.571), or ocular allergy (*p* = 0.502).

**Table 4:** Analysis of variables affecting ^1^Delta LogMAR.

Univariate analysis of continuous variables				
Variables	^2^ *N*	Pearson Correlation (^3^r)		*p*-value
Age	123	0.058		0.527
Follow-up	123	−0.127		0.164
Pachymetry	120	0.017		0.853
^4^LogMAR_pre_	123	−0.596		<0.001**
^5^Kmax_pre_	123	−0.034		0.712
^6^Cyl_pre_	114	−0.036		0.701
Univariate analysis of categorical variables				
Variables	*N*	Mean	Median	*p*-value
Accelerated	83 40	−0.04 −0.07	0 −0.07	0.350
Non-Accelerated				
Male	97 26	−0.04 −0.06	−0.05 −0.05	0.663
Female				
Jewish	95 26	−0.05 −0.03	−0.05 −0.04	0.571
Arab				
Allergy	35 88	−0.03 −0.05	0 −0.05	0.502
No allergy				

Univariate analysis was done with correlation analysis for continuous variables and Chi-square test for categorical variables.

1 Delta LogMAR = (LogMAR after cross-linking) – (LogMAR before cross-linking).

2 *N* = number of patients.

3 r = Pearson correlation coefficient.

4 LogMAR_pre_ = Logarithm of minimal angle of resolution before cross-linking.

5 Kmax_pre_ = maximal corneal power before cross-linking.

6 Cyl_pre_ = refractive cylinder before cross-linking.

* *p* < 0.05.

** *p* < 0.01. Bold values are for *p*-values of significance.

As only one variable was associated with a significant change in Delta LogMAR, the multivariate analysis was not relevant. These results demonstrate that lower preoperative visual acuity only influences the improvement of visual function following CXL in pediatric patients with KC.

The percentage of patients with Delta LogMAR ≤ −0.2 (gain of two or more Snellen lines) was 24.4% in the non-accelerated protocol group and 7.1% in the accelerated protocol group (*p* = 0.006; Table 3). However, the accelerated group had a larger proportion of stable eyes with Delta LogMAR between −0.2 and +0.2 (86.9% vs. 60.1% in the non-accelerated group; *p* = 0.008) and a non-significant trend towards a smaller proportion of eyes with Delta LogMAR ≥ +0.2 (6% vs. 14.6% in the non-accelerated group; *p* = 0.17).

## Discussion

We analyzed the effects of preoperative predictors of successful CXL in pediatric patients with progressive KC by using an extensive database of patients with a long-term follow-up period. We used two primary outcome measures: changes in Kmax and visual acuity, over the follow-up period. Our study showed that CXL in this group not only successfully stabilized KC progression over time, but the mean negative Delta Kmax and Delta LogMAR results also indicated improvement of corneal shape and visual function, respectively. In addition, to the best of our knowledge, our study is the first to demonstrate that, in a multivariate analysis, non-accelerated CXL and higher Kmax pre-CXL positively influenced corneal flattening, whereas lower preoperative visual acuity only influenced the improvement of visual function. These results suggest that CXL may have a more potent effect in children with advanced KC.

In the adult population, there is robust evidence of CXL success and long-lasting effects in long-term follow-up (24, 25). The results in the pediatric keratoconus patients are still controversial. Overall, epithelium-off studies show stability or flattening of the cornea while maintaining or improving the CDVA (7–11). However, some authors report significant KC progression post-CXL, with increasing rates over time (14–18). Kodavoor (18) reported Kmax worsening in 8.6% of treated eyes at one-year post-CXL. Godefrooji (17) reported Kmax progression of up to 7.2D in 22% of treated eyes in the last follow-up, with no significant changes in visual acuity. Padmanabhan (15) showed an increase of more than 1D in Kmax in 15% of treated eyes at 2 years, 21% at 4 years, and 24% beyond that period, with a decrease in visual acuity of 30.9% after 4 years. A similar CXL regression effect was noted by Mazzota (16), with a 24% progression rate over a 10-year follow-up period. Chatzis and Hafezi (14) reported the highest rate of progression, with 55% of treated eyes showing an increase of 1D or more after 3 years. In addition, the only randomized controlled trial (20) on this population showed KC progression in 7% of the treated eyes after 18 months. In general, the loss of CXL effect is poorly understood. While some authors may attribute this finding to eye rubbing secondary to chronic ocular allergic disease (14, 16, 18), it could be secondary to the more aggressive course of KC in this population (2, 26), the lack of natural cross-links in pediatric young corneas, and the corneal collagen turnover (16). Our results showed an increase of ≥1.0 D in Kmax in 15.8% of the eyes with a loss of ≥2 Snellen lines in 7.2%. Interestingly, in the univariate analysis, a longer follow-up period was correlated with a more negative Delta Kmax, indicating that the CXL effect was maintained or increased over time. However, no significant differences were found in multivariate analysis.

A higher Kmax_pre_ correlated with a significantly more negative delta Kmax. Although studies performed in the adult population support our findings (27, 28), reports on the pediatric population failed to establish a correlation between Kmax_pre_ and Delta Kmax (11, 17, 21). Interestingly, Padmanabhan (15) showed that the decrease in Kmax post-CXL was maximal in eyes with a moderate grade of disease than in the severe and mild groups.

The explanation for greater CXL flattening effect seen with higher Kmax_pre_ is still not clear. As far as we are concerned, the existing literature in adults, with similar findings as ours, fails to establish a clear cause for this effect. Sloot et al. (28) speculate that there “might be a difference in the corneal stroma in mild to moderate cases versus advanced cases of KC.” In addition, the authors believe that “treatment might be more deeply located in the stroma in advanced cases because these have thinner corneas.” (28) Our work shows that indeed in univariate analysis, pre-operatively thinner corneas were also correlated with a more negative Kmax. Although this was not the case in the multivariate analysis, as we explained in the manuscript, stepwise linear regression grades variables according to the strength of their correlation with the outcome. It might be the case that Kmax_pre_ correlated better with delta kmax and since steeper corneas usually are thinner, the pre-operative thickness was excluded from the multivariate analysis.

Like Uçakhan (11), we did not find an association between Kmax_pre_ and Delta LogMAR.

In univariate analysis, thinner corneas were correlated with a more negative Delta Kmax. This finding is in contrast with those of other published reports. Sarac (21) found that eyes with corneas thinner than 450 μm had an OR of 4.54 to progress after CXL. The authors believe that, in this group, the driving factors for KC progression are especially stronger in the more advanced (thinner) diseased corneas, and the use of hypotonic riboflavin during treatment in these eyes provided a weaker CXL effect. Kodavoor (18) showed that 1-year post-CXL, patients with a thinner cornea (between 350 and 400 μm) had a mean Kmax of 3 D more than those with thicker corneas (>400 μm). In contrast, while searching for predictors of KC progression post-CXL, Godefrooji (17) did not find a correlation between pachymetry and outcomes. The Siena CXL Pediatrics study (8) found that both groups of eyes with corneas > or <450 μm had statistically significant flattening of Kmax during all follow-up periods, with a similar mean decrease in Kmax. Similarly, with an accelerated protocol, Ulusoy (12) showed that keratometry and CDVA improved from baseline after 1 year in eyes with corneas < and ≥450 μm. We believe that our results better reflect clinical CXL effects because we analyzed corneal thickness in a large cohort as a continuous variable rather than using a categorical variable approach. Moreover, corneal thickness was correlated with KC severity. Thus, eyes with thinner corneas had more severe topographic characteristics (such as Kmax_pre_) at baseline. Stepwise linear regression grade variables according to the strength of their correlation with the outcome. Other parameters (Kmax_pre_) probably correlated better with the outcome; hence, pachymetry was found to be nonsignificant in the multivariate analysis. Pachymetry was not associated with logMAR changes, similar to the findings of other studies (16, 18).

Worse baseline visual acuity (higher LogMAR_pre_) was significantly correlated with corneal flattening and better visual acuity post-CXL in univariate analysis. Although studies on the adult population support our findings (29–31) data on pediatric patients contradict our results. Uçakhan (11) did not find an association between preoperative vision and improvement in uncorrected or corrected distance visual acuity in 40 eyes during 48 months of follow-up. While evaluating predictive factors for the progression of KC post-CXL, Sarac (21) and Godefrooij (17) did not find a significant effect of CDVA on an increase in Kmax ≥1 D. Interestingly, despite the usual vision worsening with KC progression, some studies have reported no vision change with an increase in Kmax post-CXL (14, 17). In a previous work by Wisse et al. (31), worse baseline visual acuity correlated with more central cones, which demonstrated greater CXL effect, presumably due to a more perpendicular incident angle of UVA rays, as compared with more peripheral cones. However, we did not evaluate cone eccentricity in our study. It seems that further studies are needed to elucidate the mechanism explaining the observation of greater visual improvement with worse baseline visual acuity.

Non-accelerated CXL correlated with significant Kmax flattening compared with accelerated CXL. Amer (32) found that the CDVA and Kmax improved more in the non-accelerated group. Turhan (33) found a significant decrease in Kmax in both accelerated and non-accelerated groups. However, after 2 years, CDVA only improved from baseline in the non-accelerated group. Sarac (34) found no difference in the outcomes between the non-accelerated and accelerated groups. However, the post-CXL progression rates in the non-accelerated and accelerated CXL groups were 13.1 and 16.3%, respectively (*p* = 0.754).

Although the exact mechanism explaining superior efficacy of the non-accelerated protocol is not clear to us, a previous study on porcine corneas found non-accelerated CXL to produce greater anterior stromal stiffening, as compared to the accelerated protocol (35).

In contrast, a prospective randomized contralateral eye study (36) showed better improvement in vision and Kmax with the accelerated protocol after 3 years. The authors stated that conventional and accelerated CXL are effective options for managing KC in children. Accelerated CXL is more appealing to both doctors and patients because of its shorter duration and, therefore, better patient comfort and compliance during local anesthesia. However, we believe that the benefits of non-accelerated treatment outweigh its disadvantages, with a significantly more potent effect characterized by 95 vs. 79% stabilization or flattening of Kmax in children.

Age did not affect any of the outcome measures in this study. Other studies (11, 17, 21) also failed to demonstrate a correlation with age. However, Mazzotta (16). observed that a treatment regression rate of 24% over 10 years could be considered for patients aged 15 years at the time of treatment. When evaluating the accelerated CXL protocol, Ozer (19) found keratometric progression in 35% of patients aged ≤14 years and 4% of patients aged 15–18 years (*p* = 0.014). Although some may argue that younger patients have more severe allergic eye diseases and are less compliant with ceasing eye rubbing (19), in addition to having more aggressive KC (2–4), we believe that our results are consistent because age was analyzed as a continuous variable (and not dichotomously, as in those studies).

Our previous work evaluated success predictors of CXL in adult KC in 517 eyes, with a mean followed for 2.3 years (37). The ethnic populations were similar to the present study in children. In resemblance with the present study, Delta Kmax was influenced negatively by a longer follow-up, thinner corneas, non-accelerated protocol, higher Kmaxpre and higher LogMARpre in univariate analysis and non-accelerated protocol and higher Kmax in multivariate analysis. Similarly, the only significant feature when analyzing the Delta LogMAR was a higher LogMARpre. The results of both studies corroborate the idea that CXL effect is higher in more advanced diseases regardless of age and that the non-accelerated protocol is more effective than the accelerated CXL. In addition, although pediatric corneas have fewer natural cross-links and are subjected to more aggressive disease, CXL treatment behaves alike.

Allergy was not associated with changes in the CDVA and Kmax. Previous studies have linked allergy and eye rubbing to CXL failure or regression (14, 18). KC is more severe and progresses faster in patients with VKC and ocular allergies than in those without VKC (38).Nonetheless, in a study comparing CXL in pediatric patients with KC with and without VKC, there were no differences in outcome, complications, or KC progression between the groups (39).

Middle Eastern populations are known to have a greater incidence and severity of KC (3). Nevertheless, our study did not find a correlation between Jewish or Arabic origin and CXL success in Israel. However, the pathogenesis of KC is not yet completely understood. Some KC features suggest a strong genetic component (40). Our study population comprised both the Jewish and Arabic populations of Jerusalem and its surroundings. High rates of consanguineous marriages characterize these two subsets of the local population; therefore, genetic diseases are relatively more common. We believe that similar marital habits might explain the similarities in the outcomes between the two groups.

Our study has significant advantages over the existing literature. First, the sample size was significantly larger than those reported in previous studies. Second, we followed the patients for an extended period of up to 8 years. Third, many variables in this study were analyzed as continuous (Kmax, LogMAR, age, CCT, and follow-up) and not categorically as in previous studies. Fourth, we included the effects of allergic eye disease and ethnicity on the CXL outcomes. Finally, this is one of the few studies to present a systematic analysis of multiple predictors of CXL success using univariate and multivariate statistical tools.

Our study has some limitations. This was a retrospective analysis; therefore, some data were missing from a small proportion of the cohort. Second, the study cohort lacked control eyes that had not been treated with CXL. However, as the efficacy of CXL has been demonstrated in many previous studies, it is unethical to not perform this procedure in patients with progressive KC. In addition, our analysis included cases from 2007, when CXL was less popular and well known and was not yet introduced to the Israeli National Health basket until 2014. This means that cases performed in the early years might have had a more advanced KC, whereas those performed in the later years might have had a more moderate KC. As the procedure gained acceptance and popularity, and with the growing awareness of its efficacy, more community ophthalmologists and optometrists referred more patients with KC at earlier stages. Lastly, it mainly comprises Middle Eastern patients, who are known to have a more aggressive progression of KC than other populations (3).

In conclusion, our results show that CXL is an effective treatment for decrease in Kmax and to improve vision in pediatric patients with KC. Patients with an advanced KC may benefit more from the late effects of CXL, with a decrease in Kmax, than patients with early KC do. However, early detection and treatment are critical for patient benefit, and treatment should not be delayed to achieve maximum effects. Finally, we demonstrated that the non-accelerated protocol had a greater impact on corneal flattening than the accelerated protocol did.

## Data availability statement

The raw data supporting the conclusions of this article will be made available by the authors, without undue reservation.

## Ethics statement

The studies involving human participants were reviewed and approved by this study adhered to the principles of the Declaration of Helsinki. Approval was obtained from the Institutional Review (IRB)Board/Ethics Committee of Hadassah medical center, Jerusalem, Israel (approval number 18-0221). Written informed consent from the participants’ legal guardian/next of kin was not required to participate in this study in accordance with the national legislation and the institutional requirements.

## Author contributions

DW, JF-P, and AS: conceptualization. DW, OS, YT, JF-P, and AS: data curation and formal analysis. DW, OS, JF-P, and AS: methodology. AS: project administration, resources, and supervision. YT and AS: validation. DW, OS, YT, and AS: writing–original draft. DW, OS, and AS: writing—review and editing. All authors contributed to the article and approved the submitted version.

## Conflict of interest

The authors declare that the research was conducted in the absence of any commercial or financial relationships that could be construed as a potential conflict of interest.

## Publisher’s note

All claims expressed in this article are solely those of the authors and do not necessarily represent those of their affiliated organizations, or those of the publisher, the editors and the reviewers. Any product that may be evaluated in this article, or claim that may be made by its manufacturer, is not guaranteed or endorsed by the publisher.

## Abbreviations

CDVA: Corrected distance visual acuity
Kmax: Maximal corneal curvature
Kmax_pre_: Maximal corneal curvature before cross-linking
Kmax_last_: Maximal corneal curvature after cross-linking at last follow up
LogMAR_pre_: Logarithm of minimal angle of resolution
LogMAR_pre_: Logarithm of minimal angle of resolution before cross-linking
LogMAR_last_: Logarithm of minimal angle of resolution after cross-linking at last follow up
Cyl_pre_: Refractive cylinder before cross-linking
CCT_pre_: Central corneal thickness before cross linking
SE_pre_: Spherical equivalent before cross-linking
CXL: Cross-linking
KC: Keratoconus
SD: Standard deviation

## References

[1] Yang K Gu Y Xu L Fan Q Zhu M Wang Q. Distribution of pediatric keratoconus by different age and gender groups. Front Pediatr. (2022) 10:937246. doi: 10.3389/fped.2022.937246, PMID: 35923788 PMC9339668

[2] Léoni-Mesplié S Mortemousque B Touboul D Malet F Praud D Mesplié N. Scalability and severity of keratoconus in children. Am J Ophthalmol. (2012) 154:56–62.e1. doi: 10.1016/j.ajo.2012.01.025, PMID: 22534107

[3] Ferdi AC Nguyen V Gore DM Allan BD Rozema JJ Watson SL. Keratoconus natural progression: a systematic review and meta-analysis of 11 529 eyes. Ophthalmology. (2019) 126:935–45. doi: 10.1016/j.ophtha.2019.02.029 30858022

[4] Olivo-Payne A Abdala-Figuerola A Hernandez-Bogantes E Pedro-Aguilar L Chan E Godefrooij D. Optimal management of pediatric keratoconus: challenges and solutions. Clin Ophthalmol. (2019) 13:1183–91. doi: 10.2147/OPTH.S183347, PMID: 31371915 PMC6628904

[5] Wollensak G Spoerl E Seiler T. Riboflavin/ultraviolet-A-induced collagen crosslinking for the treatment of keratoconus. Am J Ophthalmol. (2003) 135:620–7. doi: 10.1016/S0002-9394(02)02220-1, PMID: 12719068

[6] Koller T Mrochen M Seiler T. Complication and failure rates after corneal crosslinking. J Cataract Refract Surg. (2009) 35:1358–62. doi: 10.1016/j.jcrs.2009.03.035, PMID: 19631120

[7] Vinciguerra P Albé E Frueh BE Trazza S Epstein D. Two-year corneal cross-linking results in patients younger than 18 years with documented progressive keratoconus. Am J Ophthalmol. (2012) 154:520–6. doi: 10.1016/j.ajo.2012.03.020, PMID: 22633357

[8] Caporossi A Mazzotta C Baiocchi S Caporossi T Denaro R Balestrazzi A. Riboflavin-UVA-induced corneal collagen cross-linking in pediatric patients. Cornea. (2012) 31:227–31. doi: 10.1097/ICO.0b013e31822159f6, PMID: 22420024

[9] Zotta PG Diakonis VF Kymionis GD Grentzelos M Moschou KA. Long-term outcomes of corneal cross-linking for keratoconus in pediatric patients. J AAPOS. (2017) 21:397–401. doi: 10.1016/j.jaapos.2017.07.205, PMID: 28935449

[10] Wise S Diaz C Termote K Dubord PJ McCarthy M Yeung SN. Corneal cross-linking in pediatric patients with progressive Keratoconus. Cornea. (2016) 35:1441–3. doi: 10.1097/ICO.0000000000000923 27310886

[11] Uçakhan ÖO Bayraktutar BN Saglik A. Pediatric corneal collagen cross-linking: long-term follow-up of visual, refractive, and topographic outcomes. Cornea. (2016) 35:162–8. doi: 10.1097/ICO.0000000000000702 26655483

[12] Ulusoy DM Göktaş E Duru N Özköse A Ataş M Yuvacı İ. Accelerated corneal crosslinking for treatment of progressive keratoconus in pediatric patients. Eur J Ophthalmol. (2017) 27:319–25. doi: 10.5301/ejo.5000848 27445064

[13] Ozgurhan EB Kara N Cankaya KI Kurt T Demirok A. Accelerated corneal cross-linking in pediatric patients with keratoconus: 24-month outcomes. J Refract Surg. (2014) 30:843–9. doi: 10.3928/1081597X-20141120-01, PMID: 25437484

[14] Chatzis N Hafezi F. Progression of keratoconus and efficacy of corneal collagen cross-linking in children and adolescents. J Refract Surg. (2012) 28:753–8. doi: 10.3928/1081597X-20121011-01 23347367

[15] Padmanabhan P Rachapalle Reddi S Rajagopal R Natarajan R Iyer G Srinivasan B. Corneal collagen cross-linking for Keratoconus in pediatric patients - long-term results. Cornea. (2017) 36:138–43. doi: 10.1097/ICO.0000000000001102, PMID: 28060058

[16] Mazzotta C Traversi C Baiocchi S Bagaglia S Caporossi O Villano A. Corneal collagen cross-linking with riboflavin and ultraviolet a light for pediatric Keratoconus: ten-year results. Cornea. (2018) 37:560–6. doi: 10.1097/ICO.0000000000001505, PMID: 29319598

[17] Godefrooij DA Soeters N Imhof SM Wisse RPL. Corneal cross-linking for pediatric Keratoconus: long-term results. Cornea. (2016) 35:954–8. doi: 10.1097/ICO.0000000000000819 27027921

[18] Kodavoor SK Arsiwala AZ Ramamurthy D. One-year clinical study on efficacy of corneal cross-linking in Indian children with progressive keratoconus. Cornea. (2014) 33:919–22. doi: 10.1097/ICO.0000000000000197, PMID: 25055145

[19] Ozer MD Batur M Mesen S Tekın S Seven E Yasar T. Comparison of the efficacy of accelerated corneal cross-linking therapy in different pediatric age groups having progressive keratoconus. Int Ophthalmol. (2020) 40:2651–8. doi: 10.1007/s10792-020-01446-w, PMID: 32488590

[20] Larkin DFP Chowdhury K Burr JM Raynor M Edwards M Tuft SJ. Effect of corneal cross-linking versus standard care on Keratoconus progression in young patients: the KERALINK randomized controlled trial. Ophthalmology. (2021) 128:1516–26. doi: 10.1016/j.ophtha.2021.04.019, PMID: 33892046

[21] Sarac O Caglayan M Cakmak HB Cagil N. Factors influencing progression of keratoconus 2 years after corneal collagen cross-linking in pediatric patients. Cornea. (2016) 35:1503–7. doi: 10.1097/ICO.0000000000001051, PMID: 27741013

[22] Maeda N Klyce SD Smolek MK. Comparison of methods for detecting Keratoconus using Videokeratography. Arch Ophthalmol. (1995) 113:870. doi: 10.1001/archopht.1995.01100070044023 7605277

[23] Koppen C Gobin L Mathysen D Wouters K Tassignon MJ. Influence of contact lens wear on the results of ultraviolet a/riboflavin cross-linking for progressive keratoconus. Br J Ophthalmol. (2011) 95:1402–5. doi: 10.1136/bjophthalmol-2011-300329, PMID: 21795286

[24] O’Brart DPS Patel P Lascaratos G Wagh VK Tam C Lee J. Corneal cross-linking to halt the progression of Keratoconus and corneal ectasia: seven-year follow-up. Am J Ophthalmol. (2015) 160:1154–63. doi: 10.1016/j.ajo.2015.08.023, PMID: 26307513

[25] Raiskup F Theuring A Pillunat LE Spoerl E. Corneal collagen crosslinking with riboflavin and ultraviolet-a light in progressive keratoconus: ten-year results. J Cataract Refract Surg. (2015) 41:41–6. doi: 10.1016/j.jcrs.2014.09.033, PMID: 25532633

[26] McMahon TT Edrington TB Szczotka-Flynn L Olafsson HE Davis LJ Schechtman KB. Longitudinal changes in corneal curvature in keratoconus. Cornea. (2006) 25:296–305. doi: 10.1097/01.ico.0000178728.57435.df, PMID: 16633030

[27] Koller T Pajic B Vinciguerra P Seiler T. Flattening of the cornea after collagen crosslinking for keratoconus. J Cataract Refract Surg. (2011) 37:1488–92. doi: 10.1016/j.jcrs.2011.03.041 21782091

[28] Sloot F Soeters N Van Der Valk R Tahzib NG. Effective corneal collagen crosslinking in advanced cases of progressive keratoconus. J Cataract Refract Surg. (2013) 39:1141–5. doi: 10.1016/j.jcrs.2013.01.045, PMID: 23711873

[29] Toprak I Yaylali V Yildirim C. Factors affecting outcomes of corneal collagen crosslinking treatment. Eye. (2014) 28:41–6. doi: 10.1038/eye.2013.224, PMID: 24136568 PMC3890756

[30] Godefrooij DA Boom K Soeters N Imhof SM Wisse RPL. Predictors for treatment outcomes after corneal crosslinking for keratoconus: a validation study. Int Ophthalmol. (2017) 37:341–8. doi: 10.1007/s10792-016-0262-z, PMID: 27221267 PMC5346429

[31] Wisse RPL Godefrooij DA Soeters N Imhof SM Van Der Lelij A. A multivariate analysis and statistical model for predicting visual acuity and keratometry one year after cross-linking for keratoconus. Am J Ophthalmol. (2014) 157:519–525.e2. doi: 10.1016/j.ajo.2013.11.001, PMID: 24211861

[32] Amer I Elaskary A Mostafa A Hazem HA Omar A Abdou A. Long-term visual, refractive and topographic outcomes of “epi-off” corneal collagen cross-linking in pediatric keratoconus: standard versus accelerated protocol. Clin Ophthalmol. (2020) 14:3747–54. doi: 10.2147/OPTH.S275797, PMID: 33177802 PMC7650037

[33] Turhan SA Yargi B Toker E. Efficacy of conventional versus accelerated corneal cross-linking in pediatric keratoconus: two-year outcomes. J Refract Surg. (2020) 36:265–9. doi: 10.3928/1081597X-20200302-01, PMID: 32267958

[34] Sarac O Caglayan M Uysal BS Uzel AGT Tanriverdi B Cagil N. Accelerated versus standard corneal collagen cross-linking in pediatric keratoconus patients: 24 months follow-up results. Contact Lens Anterior Eye. (2018) 41:442–7. doi: 10.1016/j.clae.2018.06.001, PMID: 29910023

[35] Dias J Diakonis VF Lorenzo M Gonzalez F Porras K Douglas S. Corneal stromal elasticity and viscoelasticity assessed by atomic force microscopy after different cross linking protocols. Exp Eye Res. (2015) 138:1–5. doi: 10.1016/j.exer.2015.06.015, PMID: 26093276 PMC4553073

[36] Eissa SA Yassin A. Prospective, randomized contralateral eye study of accelerated and conventional corneal cross-linking in pediatric keratoconus. Int Ophthalmol. (2019) 39:971–9. doi: 10.1007/s10792-018-0898-y, PMID: 29564806

[37] Wajnsztajn D Shmueli O Zur K Frucht-Pery J Solomon A. Predicting factors for the efficacy of crosslinking for keratoconus. PLoS One. (2022) 17:e0263528. doi: 10.1371/journal.pone.0263528, PMID: 35113959 PMC8812864

[38] Wajnsztajn DSA. Vernal keratoconjunctivitis and keratoconus. Curr Opin Allergy Clin Immunol. (2021) 21:507–14. doi: 10.1097/ACI.0000000000000765 34269743

[39] Alrobaian M Elsayed M Alotaibi A Alharbi M May W Stone D. Safety and efficacy of corneal cross-linking in pediatric patients with keratoconus and vernal keratoconjunctivitis. Middle East Afr J Ophthalmol. (2019) 26:95–100. doi: 10.4103/meajo.MEAJO_240_18, PMID: 31543667 PMC6737785

[40] Tuft SJ Hassan H George S Frazer DG Willoughby CE Liskova P. Keratoconus in 18 pairs of twins. Acta Ophthalmol. (2012) 90:e482–6. doi: 10.1111/j.1755-3768.2012.02448.x, PMID: 22682160

